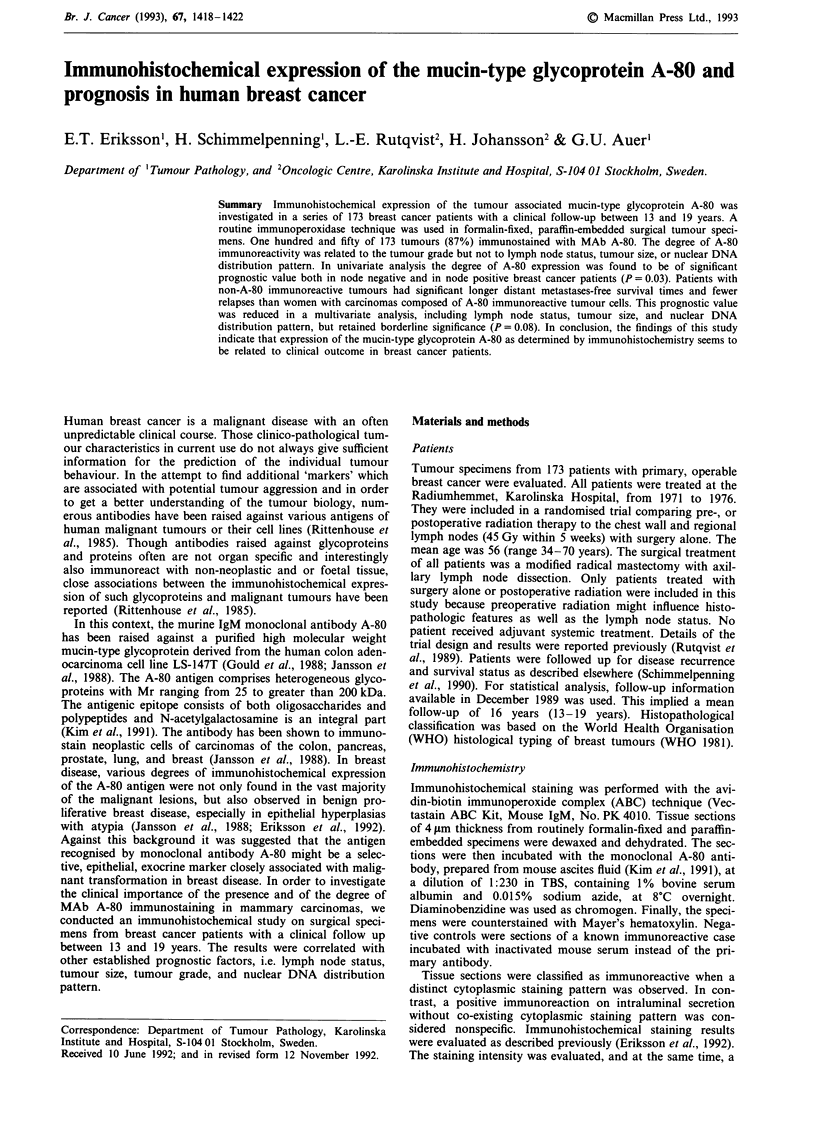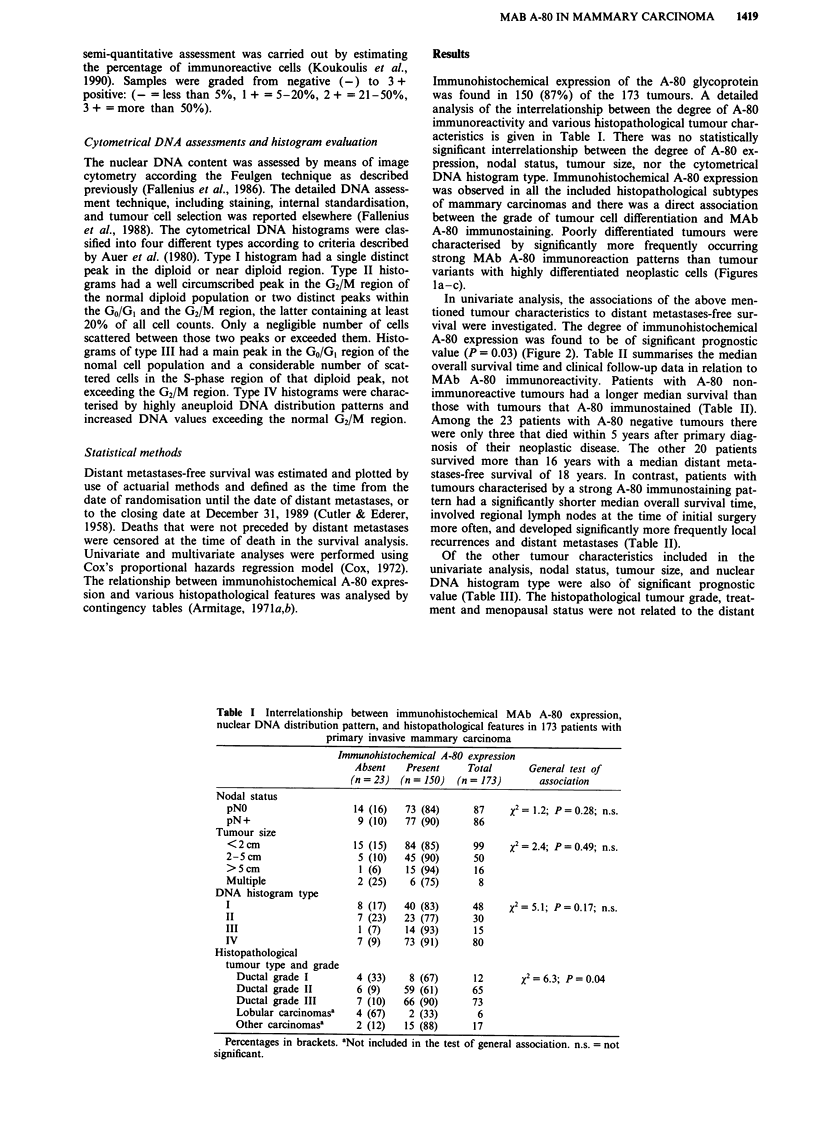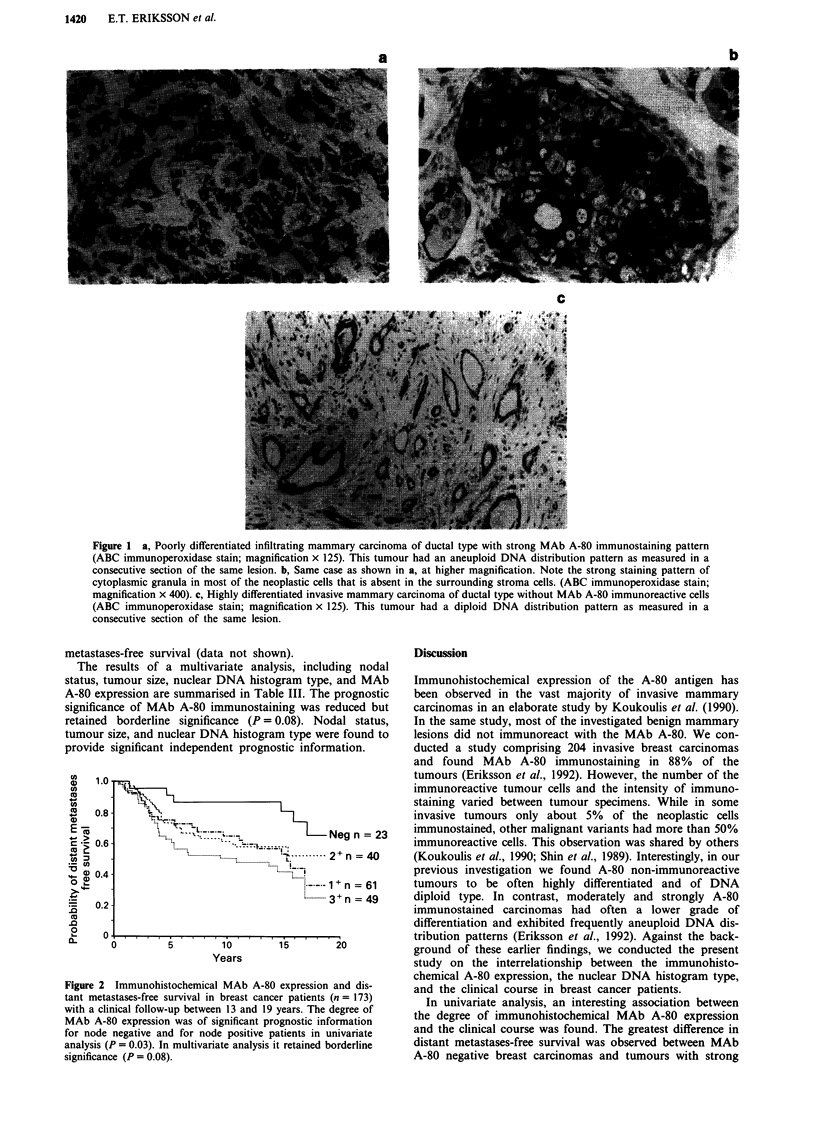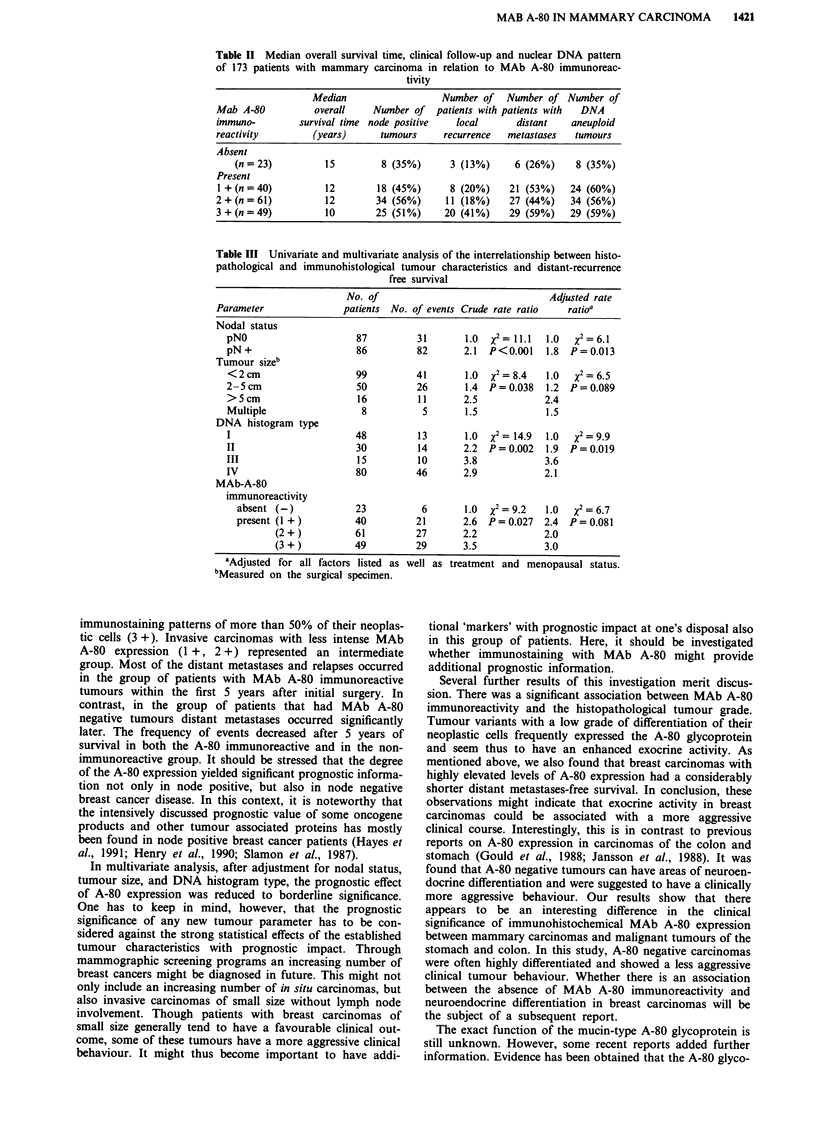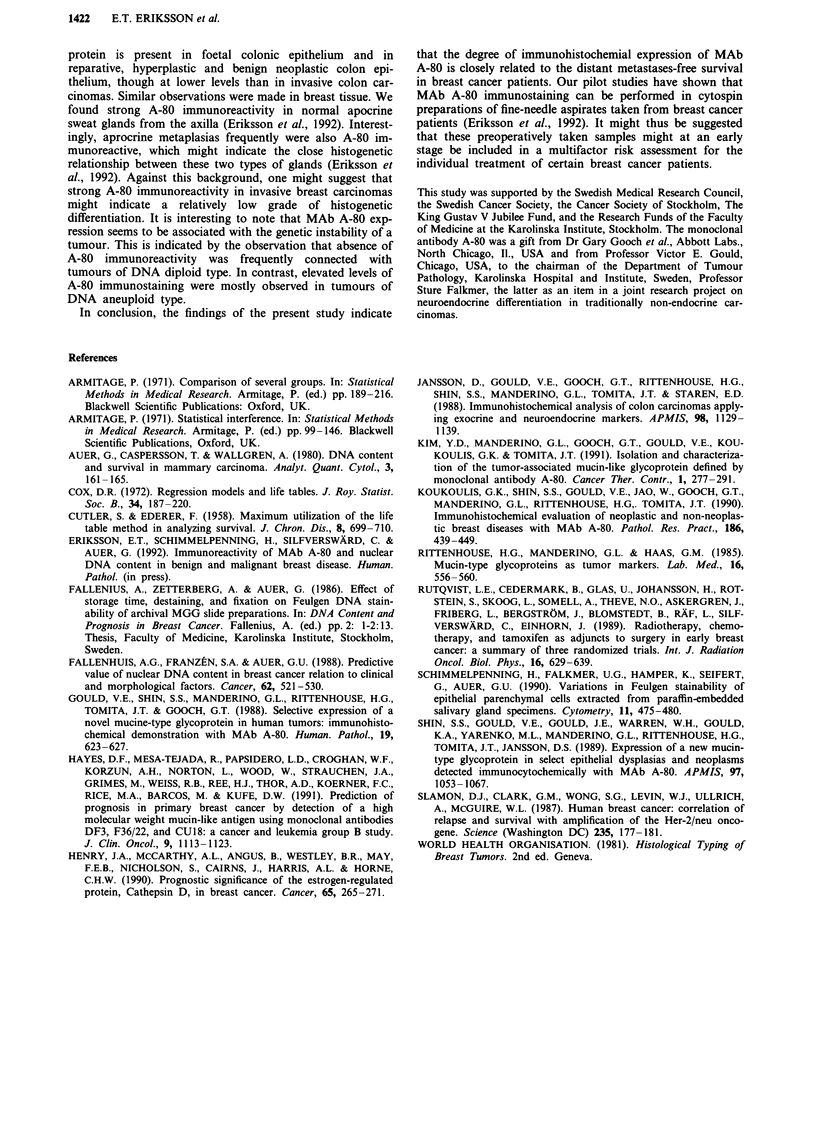# Immunohistochemical expression of the mucin-type glycoprotein A-80 and prognosis in human breast cancer.

**DOI:** 10.1038/bjc.1993.263

**Published:** 1993-06

**Authors:** E. T. Eriksson, H. Schimmelpenning, L. E. Rutqvist, H. Johansson, G. U. Auer

**Affiliations:** Department of Tumour Pathology, Karolinska Institute and Hospital, Stockholm, Sweden.

## Abstract

**Images:**


Br. J. Cancer (1993), 67, 1418-1422 © Macmillan Press Ltd., 1993

### Immunohistochemical expression of the mucin-type glycoprotein A-80 and prognosis in human breast cancer

E.T. Eriksson[1], H. Schimmelpenning[1], L.-E. Rutqvist[2], H. Johansson[2] & G.U. Auer[1]

*Department of [1]Tumour Pathology, and [2]Oncologic Centre, Karolinska Institute and Hospital, S-104 01 Stockholm, Sweden.*

**Summary** Immunohistochemical expression of the tumour associated mucin-type glycoprotein A-80 was investigated in a series of 173 breast cancer patients with a clinical follow-up between 13 and 19 years. A routine immunoperoxidase technique was used in formalin-fixed, paraffin-embedded surgical tumour specimens. One hundred and fifty of 173 tumours (87%) immunostained with MAb A-80. The degree of A-80 immunoreactivity was related to the tumour grade but not to lymph node status, tumour size, or nuclear DNA distribution pattern. In univariate analysis the degree of A-80 expression was found to be of significant prognostic value both in node negative and in node positive breast cancer patients ($P = 0.03$). Patients with non-A-80 immunoreactive tumours had significant longer distant metastases-free survival times and fewer relapses than women with carcinomas composed of A-80 immunoreactive tumour cells. This prognostic value was reduced in a multivariate analysis, including lymph node status, tumour size, and nuclear DNA distribution pattern, but retained borderline significance ($P = 0.08$). In conclusion, the findings of this study indicate that expression of the mucin-type glycoprotein A-80 as determined by immunohistochemistry seems to be related to clinical outcome in breast cancer patients.

Human breast cancer is a malignant disease with an often unpredictable clinical course. Those clinico-pathological tumour characteristics in current use do not always give sufficient information for the prediction of the individual tumour behaviour. In the attempt to find additional 'markers' which are associated with potential tumour aggression and in order to get a better understanding of the tumour biology, numerous antibodies have been raised against various antigens of human malignant tumours or their cell lines (Rittenhouse *et al.*, 1985). Though antibodies raised against glycoproteins and proteins often are not organ specific and interestingly also immunoreact with non-neoplastic and or foetal tissue, close associations between the immunohistochemical expression of such glycoproteins and malignant tumours have been reported (Rittenhouse *et al.*, 1985).

In this context, the murine IgM monoclonal antibody A-80 has been raised against a purified high molecular weight mucin-type glycoprotein derived from the human colon adenocarcinoma cell line LS-147T (Gould *et al.*, 1988; Jansson *et al.*, 1988). The A-80 antigen comprises heterogeneous glycoproteins with Mr ranging from 25 to greater than 200 kDa. The antigenic epitope consists of both oligosaccharides and polypeptides and N-acetylgalactosamine is an integral part (Kim *et al.*, 1991). The antibody has been shown to immunostain neoplastic cells of carcinomas of the colon, pancreas, prostate, lung, and breast (Jansson *et al.*, 1988). In breast disease, various degrees of immunohistochemical expression of the A-80 antigen were not only found in the vast majority of the malignant lesions, but also observed in benign proliferative breast disease, especially in epithelial hyperplasias with atypia (Jansson *et al.*, 1988; Eriksson *et al.*, 1992). Against this background it was suggested that the antigen recognised by monoclonal antibody A-80 might be a selective, epithelial, exocrine marker closely associated with malignant transformation in breast disease. In order to investigate the clinical importance of the presence and of the degree of MAb A-80 immunostaining in mammary carcinomas, we conducted an immunohistochemical study on surgical specimens from breast cancer patients with a clinical follow up between 13 and 19 years. The results were correlated with other established prognostic factors, i.e. lymph node status, tumour size, tumour grade, and nuclear DNA distribution pattern.

Correspondence: Department of Tumour Pathology, Karolinska Institute and Hospital, S-104 01 Stockholm, Sweden.
Received 10 June 1992; and in revised form 12 November 1992.

### Materials and methods

### *Patients*

Tumour specimens from 173 patients with primary, operable breast cancer were evaluated. All patients were treated at the Radiumhemmet, Karolinska Hospital, from 1971 to 1976. They were included in a randomised trial comparing pre-, or postoperative radiation therapy to the chest wall and regional lymph nodes (45 Gy within 5 weeks) with surgery alone. The mean age was 56 (range 34-70 years). The surgical treatment of all patients was a modified radical mastectomy with axillary lymph node dissection. Only patients treated with surgery alone or postoperative radiation were included in this study because preoperative radiation might influence histopathologic features as well as the lymph node status. No patient received adjuvant systemic treatment. Details of the trial design and results were reported previously (Rutqvist *et al.*, 1989). Patients were followed up for disease recurrence and survival status as described elsewhere (Schimmelpenning *et al.*, 1990). For statistical analysis, follow-up information available in December 1989 was used. This implied a mean follow-up of 16 years (13-19 years). Histopathological classification was based on the World Health Organisation (WHO) histological typing of breast tumours (WHO 1981).

### *Immunohistochemistry*

Immunohistochemical staining was performed with the avidin-biotin immunoperoxide complex (ABC) technique (Vectastain ABC Kit, Mouse IgM, No. PK 4010. Tissue sections of 4 μm thickness from routinely formalin-fixed and paraffin-embedded specimens were dewaxed and dehydrated. The sections were then incubated with the monoclonal A-80 antibody, prepared from mouse ascites fluid (Kim *et al.*, 1991), at a dilution of 1:230 in TBS, containing 1% bovine serum albumin and 0.015% sodium azide, at 8°C overnight. Diaminobenzidine was used as chromogen. Finally, the specimens were counterstained with Mayer's hematoxylin. Negative controls were sections of a known immunoreactive case incubated with inactivated mouse serum instead of the primary antibody.

Tissue sections were classified as immunoreactive when a distinct cytoplasmic staining pattern was observed. In contrast, a positive immunoreaction on intraluminal secretion without co-existing cytoplasmic staining pattern was considered nonspecific. Immunohistochemical staining results were evaluated as described previously (Eriksson *et al.*, 1992). The staining intensity was evaluated, and at the same time, a semi-quantitative assessment was carried out by estimating the percentage of immunoreactive cells (Koukoulis *et al.*, 1990). Samples were graded from negative (−) to 3+ positive: (− = less than 5%, 1+ = 5–20%, 2+ = 21–50%, 3+ = more than 50%).

*Cytometrical DNA assessments and histogram evaluation*

The nuclear DNA content was assessed by means of image cytometry according the Feulgen technique as described previously (Fallenius *et al.*, 1986). The detailed DNA assessment technique, including staining, internal standardisation, and tumour cell selection was reported elsewhere (Fallenius *et al.*, 1988). The cytometrical DNA histograms were classified into four different types according to criteria described by Auer *et al.* (1980). Type I histogram had a single distinct peak in the diploid or near diploid region. Type II histograms had a well circumscribed peak in the $G_2/M$ region of the normal diploid population or two distinct peaks within the $G_0/G_1$ and the $G_2/M$ region, the latter containing at least 20% of all cell counts. Only a negligible number of cells scattered between those two peaks or exceeded them. Histograms of type III had a main peak in the $G_0/G_1$ region of the nomal cell population and a considerable number of scattered cells in the S-phase region of that diploid peak, not exceeding the $G_2/M$ region. Type IV histograms were characterised by highly aneuploid DNA distribution patterns and increased DNA values exceeding the normal $G_2/M$ region.

*Statistical methods*

Distant metastases-free survival was estimated and plotted by use of actuarial methods and defined as the time from the date of randomisation until the date of distant metastases, or to the closing date at December 31, 1989 (Cutler & Ederer, 1958). Deaths that were not preceded by distant metastases were censored at the time of death in the survival analysis. Univariate and multivariate analyses were performed using Cox's proportional hazards regression model (Cox, 1972). The relationship between immunohistochemical A-80 expression and various histopathological features was analysed by contingency tables (Armitage, 1971*a*,*b*).

### Results

Immunohistochemical expression of the A-80 glycoprotein was found in 150 (87%) of the 173 tumours. A detailed analysis of the interrelationship between the degree of A-80 immunoreactivity and various histopathological tumour characteristics is given in Table I. There was no statistically significant interrelationship between the degree of A-80 expression, nodal status, tumour size, nor the cytometrical DNA histogram type. Immunohistochemical A-80 expression was observed in all the included histopathological subtypes of mammary carcinomas and there was a direct association between the grade of tumour cell differentiation and MAb A-80 immunostaining. Poorly differentiated tumours were characterised by significantly more frequently occurring strong MAb A-80 immunoreaction patterns than tumour variants with highly differentiated neoplastic cells (Figures 1a–c).

In univariate analysis, the associations of the above mentioned tumour characteristics to distant metastases-free survival were investigated. The degree of immunohistochemical A-80 expression was found to be of significant prognostic value ($P = 0.03$) (Figure 2). Table II summarises the median overall survival time and clinical follow-up data in relation to MAb A-80 immunoreactivity. Patients with A-80 non-immunoreactive tumours had a longer median survival than those with tumours that A-80 immunostained (Table II). Among the 23 patients with A-80 negative tumours there were only three that died within 5 years after primary diagnosis of their neoplastic disease. The other 20 patients survived more than 16 years with a median distant metastases-free survival of 18 years. In contrast, patients with tumours characterised by a strong A-80 immunostaining pattern had a significantly shorter median overall survival time, involved regional lymph nodes at the time of initial surgery more often, and developed significantly more frequently local recurrences and distant metastases (Table II).

Of the other tumour characteristics included in the univariate analysis, nodal status, tumour size, and nuclear DNA histogram type were also of significant prognostic value (Table III). The histopathological tumour grade, treatment and menopausal status were not related to the distant

**Table I** Interrelationship between immunohistochemical MAb A-80 expression, nuclear DNA distribution pattern, and histopathological features in 173 patients with primary invasive mammary carcinoma

| | *Immunohistochemical A-80 expression* | | | |
|---|---|---|---|---|
| | *Absent (n = 23)* | *Present (n = 150)* | *Total (n = 173)* | *General test of association* |
| Nodal status | | | | |
| pN0 | 14 (16) | 73 (84) | 87 | $\chi^2 = 1.2$; $P = 0.28$; n.s. |
| pN+ | 9 (10) | 77 (90) | 86 | |
| Tumour size | | | | |
| <2 cm | 15 (15) | 84 (85) | 99 | $\chi^2 = 2.4$; $P = 0.49$; n.s. |
| 2–5 cm | 5 (10) | 45 (90) | 50 | |
| >5 cm | 1 (6) | 15 (94) | 16 | |
| Multiple | 2 (25) | 6 (75) | 8 | |
| DNA histogram type | | | | |
| I | 8 (17) | 40 (83) | 48 | $\chi^2 = 5.1$; $P = 0.17$; n.s. |
| II | 7 (23) | 23 (77) | 30 | |
| III | 1 (7) | 14 (93) | 15 | |
| IV | 7 (9) | 73 (91) | 80 | |
| Histopathological tumour type and grade | | | | |
| Ductal grade I | 4 (33) | 8 (67) | 12 | $\chi^2 = 6.3$; $P = 0.04$ |
| Ductal grade II | 6 (9) | 59 (61) | 65 | |
| Ductal grade III | 7 (10) | 66 (90) | 73 | |
| Lobular carcinomas[a] | 4 (67) | 2 (33) | 6 | |
| Other carcinomas[a] | 2 (12) | 15 (88) | 17 | |

Percentages in brackets. [a]Not included in the test of general association. n.s. = not significant.

**Figure 1** a, Poorly differentiated infiltrating mammary carcinoma of ductal type with strong MAb A-80 immunostaining pattern (ABC immunoperoxidase stain; magnification × 125). This tumour had an aneuploid DNA distribution pattern as measured in a consecutive section of the same lesion. b, Same case as shown in a, at higher magnification. Note the strong staining pattern of cytoplasmic granula in most of the neoplastic cells that is absent in the surrounding stroma cells. (ABC immunoperoxidase stain; magnification × 400). c, Highly differentiated invasive mammary carcinoma of ductal type without MAb A-80 immunoreactive cells (ABC immunoperoxidase stain; magnification × 125). This tumour had a diploid DNA distribution pattern as measured in a consecutive section of the same lesion.

metastases-free survival (data not shown).

The results of a multivariate analysis, including nodal status, tumour size, nuclear DNA histogram type, and MAb A-80 expression are summarised in Table III. The prognostic significance of MAb A-80 immunostaining was reduced but retained borderline significance ($P = 0.08$). Nodal status, tumour size, and nuclear DNA histogram type were found to provide significant independent prognostic information.

**Figure 2** Immunohistochemical MAb A-80 expression and distant metastases-free survival in breast cancer patients ($n = 173$) with a clinical follow-up between 13 and 19 years. The degree of MAb A-80 expression was of significant prognostic information for node negative and for node positive patients in univariate analysis ($P = 0.03$). In multivariate analysis it retained borderline significance ($P = 0.08$).

### Discussion

Immunohistochemical expression of the A-80 antigen has been observed in the vast majority of invasive mammary carcinomas in an elaborate study by Koukoulis *et al.* (1990). In the same study, most of the investigated benign mammary lesions did not immunoreact with the MAb A-80. We conducted a study comprising 204 invasive breast carcinomas and found MAb A-80 immunostaining in 88% of the tumours (Eriksson *et al.*, 1992). However, the number of the immunoreactive tumour cells and the intensity of immunostaining varied between tumour specimens. While in some invasive tumours only about 5% of the neoplastic cells immunostained, other malignant variants had more than 50% immunoreactive cells. This observation was shared by others (Koukoulis *et al.*, 1990; Shin *et al.*, 1989). Interestingly, in our previous investigation we found A-80 non-immunoreactive tumours to be often highly differentiated and of DNA diploid type. In contrast, moderately and strongly A-80 immunostained carcinomas had often a lower grade of differentiation and exhibited frequently aneuploid DNA distribution patterns (Eriksson *et al.*, 1992). Against the background of these earlier findings, we conducted the present study on the interrelationship between the immunohistochemical A-80 expression, the nuclear DNA histogram type, and the clinical course in breast cancer patients.

In univariate analysis, an interesting association between the degree of immunohistochemical MAb A-80 expression and the clinical course was found. The greatest difference in distant metastases-free survival was observed between MAb A-80 negative breast carcinomas and tumours with strong

**Table II** Median overall survival time, clinical follow-up and nuclear DNA pattern of 173 patients with mammary carcinoma in relation to MAb A-80 immunoreactivity

| *Mab A-80 immuno-reactivity* | *Median overall survival time (years)* | *Number of node positive tumours* | *Number of patients with local recurrence* | *Number of patients with distant metastases* | *Number of DNA aneuploid tumours* |
|---|---|---|---|---|---|
| *Absent* | | | | | |
| ($n = 23$) | 15 | 8 (35%) | 3 (13%) | 6 (26%) | 8 (35%) |
| *Present* | | | | | |
| 1 + ($n = 40$) | 12 | 18 (45%) | 8 (20%) | 21 (53%) | 24 (60%) |
| 2 + ($n = 61$) | 12 | 34 (56%) | 11 (18%) | 27 (44%) | 34 (56%) |
| 3 + ($n = 49$) | 10 | 25 (51%) | 20 (41%) | 29 (59%) | 29 (59%) |

**Table III** Univariate and multivariate analysis of the interrelationship between histopathological and immunohistological tumour characteristics and distant-recurrence free survival

| *Parameter* | *No. of patients* | *No. of events* | *Crude rate ratio* | | *Adjusted rate ratio*[a] | |
|---|---|---|---|---|---|---|
| Nodal status | | | | | | |
| pN0 | 87 | 31 | 1.0 | $\chi^2 = 11.1$ | 1.0 | $\chi^2 = 6.1$ |
| pN + | 86 | 82 | 2.1 | $P < 0.001$ | 1.8 | $P = 0.013$ |
| Tumour size[b] | | | | | | |
| <2 cm | 99 | 41 | 1.0 | $\chi^2 = 8.4$ | 1.0 | $\chi^2 = 6.5$ |
| 2–5 cm | 50 | 26 | 1.4 | $P = 0.038$ | 1.2 | $P = 0.089$ |
| >5 cm | 16 | 11 | 2.5 | | 2.4 | |
| Multiple | 8 | 5 | 1.5 | | 1.5 | |
| DNA histogram type | | | | | | |
| I | 48 | 13 | 1.0 | $\chi^2 = 14.9$ | 1.0 | $\chi^2 = 9.9$ |
| II | 30 | 14 | 2.2 | $P = 0.002$ | 1.9 | $P = 0.019$ |
| III | 15 | 10 | 3.8 | | 3.6 | |
| IV | 80 | 46 | 2.9 | | 2.1 | |
| MAb-A-80 immunoreactivity | | | | | | |
| absent (–) | 23 | 6 | 1.0 | $\chi^2 = 9.2$ | 1.0 | $\chi^2 = 6.7$ |
| present (1 +) | 40 | 21 | 2.6 | $P = 0.027$ | 2.4 | $P = 0.081$ |
| (2 +) | 61 | 27 | 2.2 | | 2.0 | |
| (3 +) | 49 | 29 | 3.5 | | 3.0 | |

[a]Adjusted for all factors listed as well as treatment and menopausal status. [b]Measured on the surgical specimen.

immunostaining patterns of more than 50% of their neoplastic cells (3 +). Invasive carcinomas with less intense MAb A-80 expression (1 +, 2 +) represented an intermediate group. Most of the distant metastases and relapses occurred in the group of patients with MAb A-80 immunoreactive tumours within the first 5 years after initial surgery. In contrast, in the group of patients that had MAb A-80 negative tumours distant metastases occurred significantly later. The frequency of events decreased after 5 years of survival in both the A-80 immunoreactive and in the non-immunoreactive group. It should be stressed that the degree of the A-80 expression yielded significant prognostic information not only in node positive, but also in node negative breast cancer disease. In this context, it is noteworthy that the intensively discussed prognostic value of some oncogene products and other tumour associated proteins has mostly been found in node positive breast cancer patients (Hayes *et al.*, 1991; Henry *et al.*, 1990; Slamon *et al.*, 1987).

In multivariate analysis, after adjustment for nodal status, tumour size, and DNA histogram type, the prognostic effect of A-80 expression was reduced to borderline significance. One has to keep in mind, however, that the prognostic significance of any new tumour parameter has to be considered against the strong statistical effects of the established tumour characteristics with prognostic impact. Through mammographic screening programs an increasing number of breast cancers might be diagnosed in future. This might not only include an increasing number of *in situ* carcinomas, but also invasive carcinomas of small size without lymph node involvement. Though patients with breast carcinomas of small size generally tend to have a favourable clinical outcome, some of these tumours have a more aggressive clinical behaviour. It might thus become important to have additional 'markers' with prognostic impact at one's disposal also in this group of patients. Here, it should be investigated whether immunostaining with MAb A-80 might provide additional prognostic information.

Several further results of this investigation merit discussion. There was a significant association between MAb A-80 immunoreactivity and the histopathological tumour grade. Tumour variants with a low grade of differentiation of their neoplastic cells frequently expressed the A-80 glycoprotein and seem thus to have an enhanced exocrine activity. As mentioned above, we also found that breast carcinomas with highly elevated levels of A-80 expression had a considerably shorter distant metastases-free survival. In conclusion, these observations might indicate that exocrine activity in breast carcinomas could be associated with a more aggressive clinical course. Interestingly, this is in contrast to previous reports on A-80 expression in carcinomas of the colon and stomach (Gould *et al.*, 1988; Jansson *et al.*, 1988). It was found that A-80 negative tumours can have areas of neuroendocrine differentiation and were suggested to have a clinically more aggressive behaviour. Our results show that there appears to be an interesting difference in the clinical significance of immunohistochemical MAb A-80 expression between mammary carcinomas and malignant tumours of the stomach and colon. In this study, A-80 negative carcinomas were often highly differentiated and showed a less aggressive clinical tumour behaviour. Whether there is an association between the absence of MAb A-80 immunoreactivity and neuroendocrine differentiation in breast carcinomas will be the subject of a subsequent report.

The exact function of the mucin-type A-80 glycoprotein is still unknown. However, some recent reports added further information. Evidence has been obtained that the A-80 glycoprotein is present in foetal colonic epithelium and in reparative, hyperplastic and benign neoplastic colon epithelium, though at lower levels than in invasive colon carcinomas. Similar observations were made in breast tissue. We found strong A-80 immunoreactivity in normal apocrine sweat glands from the axilla (Eriksson *et al.*, 1992). Interestingly, aprocrine metaplasias frequently were also A-80 immunoreactive, which might indicate the close histogenetic relationship between these two types of glands (Eriksson *et al.*, 1992). Against this background, one might suggest that strong A-80 immunoreactivity in invasive breast carcinomas might indicate a relatively low grade of histogenetic differentiation. It is interesting to note that MAb A-80 expression seems to be associated with the genetic instability of a tumour. This is indicated by the observation that absence of A-80 immunoreactivity was frequently connected with tumours of DNA diploid type. In contrast, elevated levels of A-80 immunostaining were mostly observed in tumours of DNA aneuploid type.

In conclusion, the findings of the present study indicate that the degree of immunohistochemial expression of MAb A-80 is closely related to the distant metastases-free survival in breast cancer patients. Our pilot studies have shown that MAb A-80 immunostaining can be performed in cytospin preparations of fine-needle aspirates taken from breast cancer patients (Eriksson *et al.*, 1992). It might thus be suggested that these preoperatively taken samples might at an early stage be included in a multifactor risk assessment for the individual treatment of certain breast cancer patients.

This study was supported by the Swedish Medical Research Council, the Swedish Cancer Society, the Cancer Society of Stockholm, The King Gustav V Jubilee Fund, and the Research Funds of the Faculty of Medicine at the Karolinska Institute, Stockholm. The monoclonal antibody A-80 was a gift from Dr Gary Gooch *et al.*, Abbott Labs., North Chicago, Il., USA and from Professor Victor E. Gould, Chicago, USA, to the chairman of the Department of Tumour Pathology, Karolinska Hospital and Institute, Sweden, Professor Sture Falkmer, the latter as an item in a joint research project on neuroendocrine differentiation in traditionally non-endocrine carcinomas.

### References

ARMITAGE, P. (1971). Comparison of several groups. In: *Statistical Methods in Medical Research*. Armitage, P. (ed.) pp. 189–216. Blackwell Scientific Publications: Oxford, UK.

ARMITAGE, P. (1971). Statistical interference. In: *Statistical Methods in Medical Research*. Armitage, P. (ed.) pp. 99–146. Blackwell Scientific Publications, Oxford, UK.

AUER, G., CASPERSSON, T. & WALLGREN, A. (1980). DNA content and survival in mammary carcinoma. *Analyt. Quant. Cytol.*, **3**, 161–165.

COX, D.R. (1972). Regression models and life tables. *J. Roy. Statist. Soc. B.*, **34**, 187–220.

CUTLER, S. & EDERER, F. (1958). Maximum utilization of the life table method in analyzing survival. *J. Chron. Dis.*, **8**, 699–710.

ERIKSSON, E.T., SCHIMMELPENNING, H., SILFVERSWÄRD, C. & AUER, G. (1992). Immunoreactivity of MAb A-80 and nuclear DNA content in benign and malignant breast disease. *Human. Pathol.* (in press).

FALLENIUS, A., ZETTERBERG, A. & AUER, G. (1986). Effect of storage time, destaining, and fixation on Feulgen DNA stainability of archival MGG slide preparations. In: *DNA Content and Prognosis in Breast Cancer*. Fallenius, A. (ed.) pp. 2: 1-2:13. Thesis, Faculty of Medicine, Karolinska Institute, Stockholm, Sweden.

FALLENHUIS, A.G., FRANZÉN, S.A. & AUER, G.U. (1988). Predictive value of nuclear DNA content in breast cancer relation to clinical and morphological factors. *Cancer*, **62**, 521–530.

GOULD, V.E., SHIN, S.S., MANDERINO, G.L., RITTENHOUSE, H.G., TOMITA, J.T. & GOOCH, G.T. (1988). Selective expression of a novel mucine-type glycoprotein in human tumors: immunohistochemical demonstration with MAb A-80. *Human. Pathol.*, **19**, 623–627.

HAYES, D.F., MESA-TEJADA, R., PAPSIDERO, L.D., CROGHAN, W.F., KORZUN, A.H., NORTON, L., WOOD, W., STRAUCHEN, J.A., GRIMES, M., WEISS, R.B., REE, H.J., THOR, A.D., KOERNER, F.C., RICE, M.A., BARCOS, M. & KUFE, D.W. (1991). Prediction of prognosis in primary breast cancer by detection of a high molecular weight mucin-like antigen using monoclonal antibodies DF3, F36/22, and CU18: a cancer and leukemia group B study. *J. Clin. Oncol.*, **9**, 1113–1123.

HENRY, J.A., MCCARTHY, A.L., ANGUS, B., WESTLEY, B.R., MAY, F.E.B., NICHOLSON, S., CAIRNS, J., HARRIS, A.L. & HORNE, C.H.W. (1990). Prognostic significance of the estrogen-regulated protein, Cathepsin D, in breast cancer. *Cancer*, **65**, 265–271.

JANSSON, D., GOULD, V.E., GOOCH, G.T., RITTENHOUSE, H.G., SHIN, S.S., MANDERINO, G.L., TOMITA, J.T. & STAREN, E.D. (1988). Immunohistochemical analysis of colon carcinomas applying exocrine and neuroendocrine markers. *APMIS*, **98**, 1129–1139.

KIM, Y.D., MANDERINO, G.L., GOOCH, G.T., GOULD, V.E., KOUKOULIS, G.K. & TOMITA, J.T. (1991). Isolation and characterization of the tumor-associated mucin-like glycoprotein defined by monoclonal antibody A-80. *Cancer Ther. Contr.*, **1**, 277–291.

KOUKOULIS, G.K., SHIN, S.S., GOULD, V.E., JAO, W., GOOCH, G.T., MANDERINO, G.L., RITTENHOUSE, H.G,. TOMITA, J.T. (1990). Immunohistochemical evaluation of neoplastic and non-neoplastic breast diseases with MAb A-80. *Pathol. Res. Pract.*, **186**, 439–449.

RITTENHOUSE, H.G., MANDERINO, G.L. & HAAS, G.M. (1985). Mucin-type glycoproteins as tumor markers. *Lab. Med.*, **16**, 556–560.

RUTQVIST, L.E., CEDERMARK, B., GLAS, U., JOHANSSON, H., ROTSTEIN, S., SKOOG, L., SOMELL, A., THEVE, N.O., ASKERGREN, J., FRIBERG, L., BERGSTRÖM, J., BLOMSTEDT, B., RÄF, L., SILFVERSWÄRD, C., EINHORN, J. (1989). Radiotherapy, chemotherapy, and tamoxifen as adjuncts to surgery in early breast cancer: a summary of three randomized trials. *Int. J. Radiation Oncol. Biol. Phys.*, **16**, 629–639.

SCHIMMELPENNING, H., FALKMER, U.G., HAMPER, K., SEIFERT, G., AUER, G.U. (1990). Variations in Feulgen stainability of epithelial parenchymal cells extracted from paraffin-embedded salivary gland specimens. *Cytometry*, **11**, 475–480.

SHIN, S.S., GOULD, V.E., GOULD, J.E., WARREN, W.H., GOULD, K.A., YARENKO, M.L., MANDERINO, G.L., RITTENHOUSE, H.G., TOMITA, J.T., JANSSON, D.S. (1989). Expression of a new mucin-type glycoprotein in select epithelial dysplasias and neoplasms detected immunocytochemically with MAb A-80. *APMIS*, **97**, 1053–1067.

SLAMON, D.J., CLARK, G.M., WONG, S.G., LEVIN, W.J., ULLRICH, A., MCGUIRE, W.L. (1987). Human breast cancer: correlation of relapse and survival with amplification of the Her-2/neu oncogene. *Science* (Washington DC) **235**, 177–181.

WORLD HEALTH ORGANISATION. (1981). *Histological Typing of Breast Tumors*. 2nd ed. Geneva.